# Novel immune‐related signature based on immune cells for predicting prognosis and immunotherapy response in clear cell renal cell carcinoma

**DOI:** 10.1002/jcla.24409

**Published:** 2022-04-20

**Authors:** Libin Zhou, Hualong Fang, Min Yin, Huimin Long, Guobin Weng

**Affiliations:** ^1^ Department of Urology The Affiliated Lihuili Hospital Ningbo University Ningbo China; ^2^ Department of Urology Ningbo Medical Centre Lihuili Hospital Ningbo China; ^3^ The First Affiliated Hospital of Nanchang Nanchang China; ^4^ Department of Urology The Affiliated Yinzhou No 2 Hospital, Ningbo University Ningbo China; ^5^ 301513 Department of Urology Ningbo Yinzhou No 2 Hospital Ningbo China

**Keywords:** clear cell renal cell carcinoma, immunotherapy, prognostic signature, tumor microenvironment

## Abstract

**Background:**

Clear cell renal cell carcinoma (ccRCC) is the most common malignant tumor of the kidney and is characterized by poor prognosis. We sought to build an immune‐related prognostic signature and investigate its relationship with immunotherapy response in ccRCC.

**Methods:**

Immune‐related genes were identified by ssGSEA and WGCNA. The prognostic signature was conducted via univariate, least absolute shrinkage and selection operator, and multivariable Cox regression analyses. Kaplan‐Meier analysis, PCA, *t*‐SNE, and ROC were used to evaluate the risk model.

**Results:**

A total of 119 immune‐related genes associated with prognosis were screened out. Six immune‐related genes (CSF1, CD5L, AIM2, TIMP3, IRF6, and HHLA2) were applied to construct a prognostic signature for KIRC. Kaplan–Meier analysis showed that patients in high‐risk group had a poorer survival outcome than in low‐risk group. The 1‐, 3‐ and 5‐year AUC of the prognostic signature was 0.754, 0.715, and 0.739, respectively. Univariate and multivariate Cox regression models demonstrated that the risk signature was an independent prognostic factor for KIRC survival. GSEA analysis suggested that the high‐risk group was concentrated on immune‐related pathways. The high‐risk group with more regulatory T‐cell infiltration showed a higher expression of immune negative regulation genes. The risk score had positively relationship with TIDE score and negatively with the response of immunotherapy. The IC50 values of axitinib, sunitinib, sorafenib, and temsirolimus were lower in the high‐risk group.

**Conclusion:**

Our study defined a robust signature that may be promising for predicting clinical outcomes and immunotherapy and targeted therapy response in ccRCC patients.

## Graphical abstract

The risk model for immnotherapy and targted therapy.

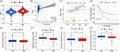

## 1 INTRODUCTION

Clear cell renal cell carcinoma (ccRCC) is the most common pathological subtype of RCC, accounting for approximately 70%–80% of RCC cases and is mainly manifested by the loss of von Hippel‐Lindau, the accumulation of lipids and glycogen and insensitivity to chemoradiotherapy. Nephrectomy is still the main treatment for ccRCC with localized disease. However, 30% of patients eventually develop into metastasis, which results in higher mortality and requires systemic treatment.^1^ In the past decade, the survival time of advanced ccRCC patients has been significantly improved due to the development of targeted and immunotherapy drugs.^2^

Immunotherapy, an important clinical program for cancer treatment that activates the immune system to attack cancer cells, is considered a promising way to treat or even cure certain cancers. Due to the unique characteristics of ccRCC, immunotherapy targeting certain components of the immune system can be applied to the clinical treatment of advanced ccRCC patients.^3^ Immune checkpoint inhibitors (ICIs) targeting the programmed cell death 1 (PD‐1), programmed cell death 1 ligand 1 (PD‐L1), and cytotoxic T lymphocyte antigen 4 (CTLA‐4) immune checkpoints have made rapid progress in ccRCC treatment. Several studies have indicated that therapeutic regimens such as nivolumab plus ipilimumab, pembrolizumab plus axitinib, and avelumab plus axitinib showed higher overall survival (OS) and objective response rates (ORRs), and they have been approved as first‐line treatments.^4^, ^5^, ^6^, ^7^, ^8^, ^9^, ^10^ With respect to efficacy, only a few people show sensitivities to immunotherapies.^11^ Therefore, how to select patient‐specific immunotherapies and combination therapies to increase response rates and decrease adverse reactions has become an important problem that might eventually be solved by further molecular biomarker stratified research for individual patients.^12^, ^13^, ^14^, ^15^

The tumor microenvironment (TME) mainly consists of tumor cells and nontumor cells, such as cancer‐associated fibroblasts (CAFs) and immune cells, which are correlated with in clinical prognosis and curative effects.^16^ Tumor‐infiltrating immune cells in the TME participate in tumor progression and immune tolerance and immune escape which can profoundly affect the response to anticancer therapies.^17^ Therefore, exploring the traits of immune cells in the KIRC TME can be helpful for immune and targeted therapy strategies.

In this study, our purpose was to uncover the potential immune‐related predictive signatures involved in ccRCC progression, prognosis, and targeted and immune‐related drug decisions by evaluating data from the Gene Expression Omnibus (GEO) and The Cancer Genome Atlas (TCGA) databases. We divided ccRCC patients into high‐ and low‐immune clusters based on the immune cells results by single sample gene set enrichment analysis (ssGSEA). Then, weighted gene co‐expression network analysis (WGCNA) was used to identify the model that was most relevant to immunity, and a six‐gene signature was established. The signature had a strong ability to forecast patient prognosis and response to targeted and ICI therapy in ccRCC.

## 2 MATERIALS AND METHODS

### 2.1 KIRC data preparation

The Series Matrix Files of GSE29609, including 39 ccRCC samples, were downloaded from GEO. The fragments per kilobase million (FPKM) values and clinical information of 539 kidney renal clear cell carcinoma (KIRC) and 72 normal samples obtained from the Genomic Data Commons (GDC, https://portal.gdc.cancer.gov/) were transformed into transcripts per kilobase million (TPM) values, which were similar to the values from GEO.^18^ The batch effects in the TCGA and GEO datasets were corrected by the “ComBat” algorithm of the sva package.^19^

### 2.2 Immune clustering based on ssGSEA

The repeated samples in TCGA were averaged and merged. Finally, 530 KIRC samples in TCGA‐KIRC and 39 KIRC samples in GEO were combined and further used for ssGSEA, which was applied to analyze the different infiltration levels of 29 kinds of immune cells and immune‐related functions in line with the levels of specific gene expression.^20^ Then, an unsupervised hierarchical clustering algorithm was performed to divide these samples into high and low‐immune clusters based on the ssGSEA results.

### 2.3 Tumor microenvironment analysis based on ESTIMATE

ESTIMATE was used to compute the scores of immune cells and stromal cells in the TME based on the expression levels of specific genes to verify the accuracy of the immune grouping using the R package “ESTIMATE.”^21^

### 2.4 GSVA for functional annotation

GSVA (gene set variation analysis) enrichment using the R package “GSVA” was applied to research the pathway differences between two clusters employing “c2.cp.kegg.v7.4.symbols” from the MSigDB database.^22^

### 2.5 Identification of the immune‐related genes (IRGs)

Differentially expressed genes between the two immune clusters were selected using the package “limma” according to |log Foldchange| > 0.5 and adjusted *p* < 0.05. Then, IRGs closely associated with the immune feature were selected from the defined differentially expressed genes by WGCNA.^23^

### 2.6 GO and KEGG function enrichment analysis

Gene Ontology (GO) enrichment and Kyoto Encyclopedia of Genes and Genomes (KEGG) pathway analysis were performed on the IRGs by R package “clusterProfiler.”^24^ After setting the criteria of adjusted *p* < 0.05, GO terms and KEGG pathways were visualized.

### 2.7 Establishment of the risk signature for KIRC

Univariate Cox regression analysis was used to identify prognostic IRGs. Next, the differentially expressed IRGs between tumor and normal samples in TCGA‐KIRC were screened out according to |logFC| > 0 and *p* < 0.05. Venn analysis was used to investigate the intersected IRGs based on the above screening conditions. Then, the slightly contributory IRGs were deleted by least absolute shrinkage and selection operator (LASSO) analysis. Finally, multivariate Cox regression analysis was applied to build an optimal prognostic signature according to the following risk formula: risk score = $\sum_{i=1}^{n}\exp i*\text{coef}i$. The terms exp*i* and coef*i* represent the expression and coefficient of the gene, respectively. The KIRC patients were assigned to high‐ and low‐risk groups based on the median risk score. The separating capacity of the risk model was further verified by principal component analysis (PCA) and *t*‐distribution stochastic neighbor embedding (*t*‐SNE). Kaplan–Meier (K‐M) and time‐dependent receiver operating characteristic (ROC) curves were utilized to evaluate the ability to predict prognosis with the risk model by the “survival” and “timeROC” R packages. Furthermore, other prognostic signatures from Gao,^25^ Wu,^26^ Zhao1,^27^ Zhao2^28^ were evaluated by ROC and C‐index.

### 2.8 Relationship between the risk model and clinical characteristics

A total of 248 patients were enrolled with complete clinical data and a follow‐up time of more than 30 days for further analysis. Univariate and multivariate Cox regression analyses were applied to confirm whether the risk score was an independent predictive variable in KIRC patients by comparing with clinical characteristics such as age, gender, stage, T stage, N stage, and M stage. The chi‐square and Wilcoxon signed‐rank tests were applied for the analysis of the relationship between the risk score and clinical characteristics. The K–M curve was used to detect the survival differences between two risk groups based on the subgroups stratified by age, gender, grade, stage, T stage, N stage, and M stage.

### 2.9 GSEA enrichment analysis

GSEA (gene set enrichment analysis) was used to analyze the GO and KEGG pathways between the high‐ and low‐risk groups in the TCGA‐KIRC database according to the gene sets “c5.go.v7.4.symbols.gmt” and “c2.cp.kegg.v7.4.symbols.gmt” by the R “clusterprofiler” package.^29^ An adjusted *p* value <0.05 represented a significant difference.

### 2.10 Immune cell infiltration in tumor microenvironment

The characteristics of immune cell infiltration in the KIRC microenvironment between the high‐ and low‐risk groups were analyzed by different software programs, such as XCELL, TIMER, QUANTISEQ, MCPCOUNTER, EPIC, CIBERSORT‐ABS, and CIBERSORT. The correlation between immune cells and risk score was shown in a lollipop diagram by Spearman correlation analysis. The Wilcoxon signed‐rank test was used to analyze the different numbers of immune cells between the two risk groups.

### 2.11 Responses to immunotherapy and targeted drug therapy

The TIDE (Tumor Immune Dysfunction and Exclusion) method (http://tide.dfci.harvard.edu/) was applied to forecast the response of KIRC patients to the immunotherapy.^30^ The RNA data and clinical information of renal cell carcinoma obtained from the IMvigor210 dataset (http://research‐pub.gene.com/IMvigor210CoreBiologies) by the “IMvigor” package were used to predict the responses of the two risk groups to the PD‐L1 inhibitor atezolizumab.^31^ Targeted drugs such as axitinib, sunitinib, sorafenib, and temsirolimus are recommended to treat ccRCC according to AJCC guidelines. To evaluate the risk model in predicting the sensitivity to targeted drugs, TCGA‐KIRC samples were applied for analysis of the IC50 between two risk groups by the “pRRophetic” package.^32^

### 2.12 Statistical analysis

R software version 4.1.2 was applied for statistical analysis. The differences between two groups were compared by the Wilcoxon signed‐rank test and analyzed by the Kruskal–Wallis test. The frequency differences were determined by the chi‐square test. Student's *t*‐test was performed to compare the expression levels in different groups when appropriate. The difference in survival was evaluated by the log‐rank test. A two‐sided *p* value <0.05 was considered statistically significant.

## 3 RESULTS

### 3.1 Establishment and validation of the immune cluster in ccRCC

**FIGURE 1:**
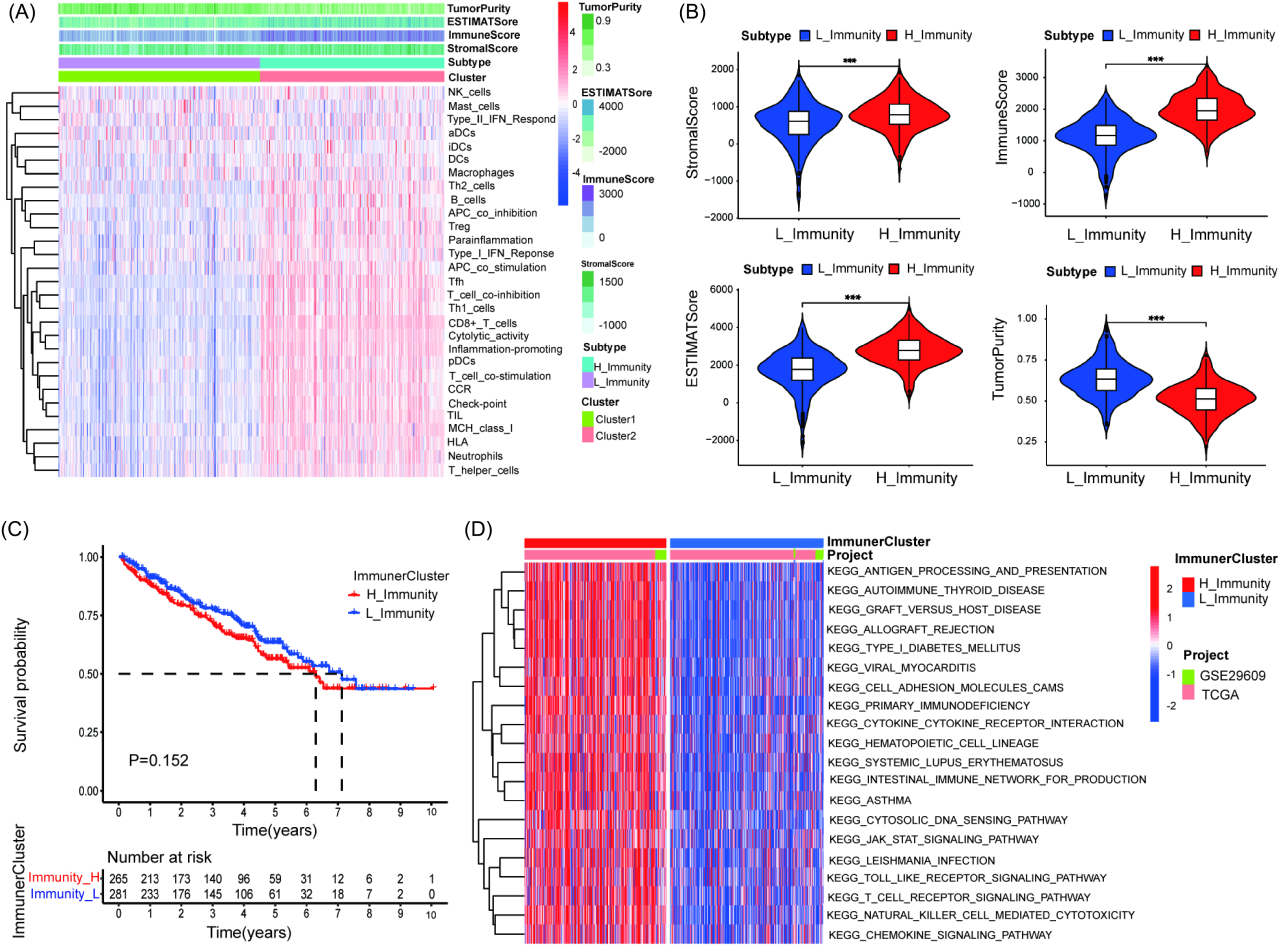
Construction and validation of immune clusters in ccRCC. (A) Differences in 29 immune‐related cells and types, tumor purity, ESTIMATE score, immune score, and stromal score between Cluster 1 (L_Immunity) and Cluster 2 (H_Immunity). (B) Violin plots showing the differences in stromal score, immune score, ESTIMATE score, and tumor purity between H_Immunity and L_Immunity. (C) Survival analysis between H_Immunity and L_Immunity. (D) GSVA enrichment analysis revealed the different activated biological pathways. ***p < 0.001

Data of 530 and 39 ccRCC samples obtained from TCGA and GSE29609 were evaluated for the immune cell infiltration by ssGSEA. Furthermore, the ccRCC samples were assigned to two clusters based on the results of 29 immune‐related cells or functions by an unsupervised hierarchical clustering algorithm. Cluster 1 (*n* = 297) was defined as the low‐immune cluster (L_Immunity) due to the characteristic of low‐immune cells (Figure 1A). Cluster 2 (*n* = 272) was defined as a high immune cluster (H_Immunity) due to the characteristic of high immune cells (Figure 1A). Then, the ESTIMATE algorithm was used to verify the application value of the immune clusters. As shown in Figure 1A, the stromal score, immune score, and ESTIMATE score in H_Immunity were higher than those in L_Immunity, while tumor purity had the opposite results. Furthermore, the similar trends were seen in the violin plots (*p* < 0.05) (Figure 1B). In addition, the prognosis of patients was not significantly different between the H_Immunity and L_Immunity groups (Figure 1C). Finally, GSVA enrichment analysis indicated that immune‐related pathways such as antigen processing and presentation, primary immunodeficiency, cytokine‐cytokine receptor interaction, intestinal immune network for IgC production, T‐cell receptor signaling pathway, natural killer cell‐mediated cytotoxicity, and chemokine signaling pathway were significantly enriched in the H_Immunity cluster (Figure 1D). All the above results revealed that the immune clusters were significantly associated with ccRCC immunity.

### 3.2 Identification of immune‐related genes

**FIGURE 2:**
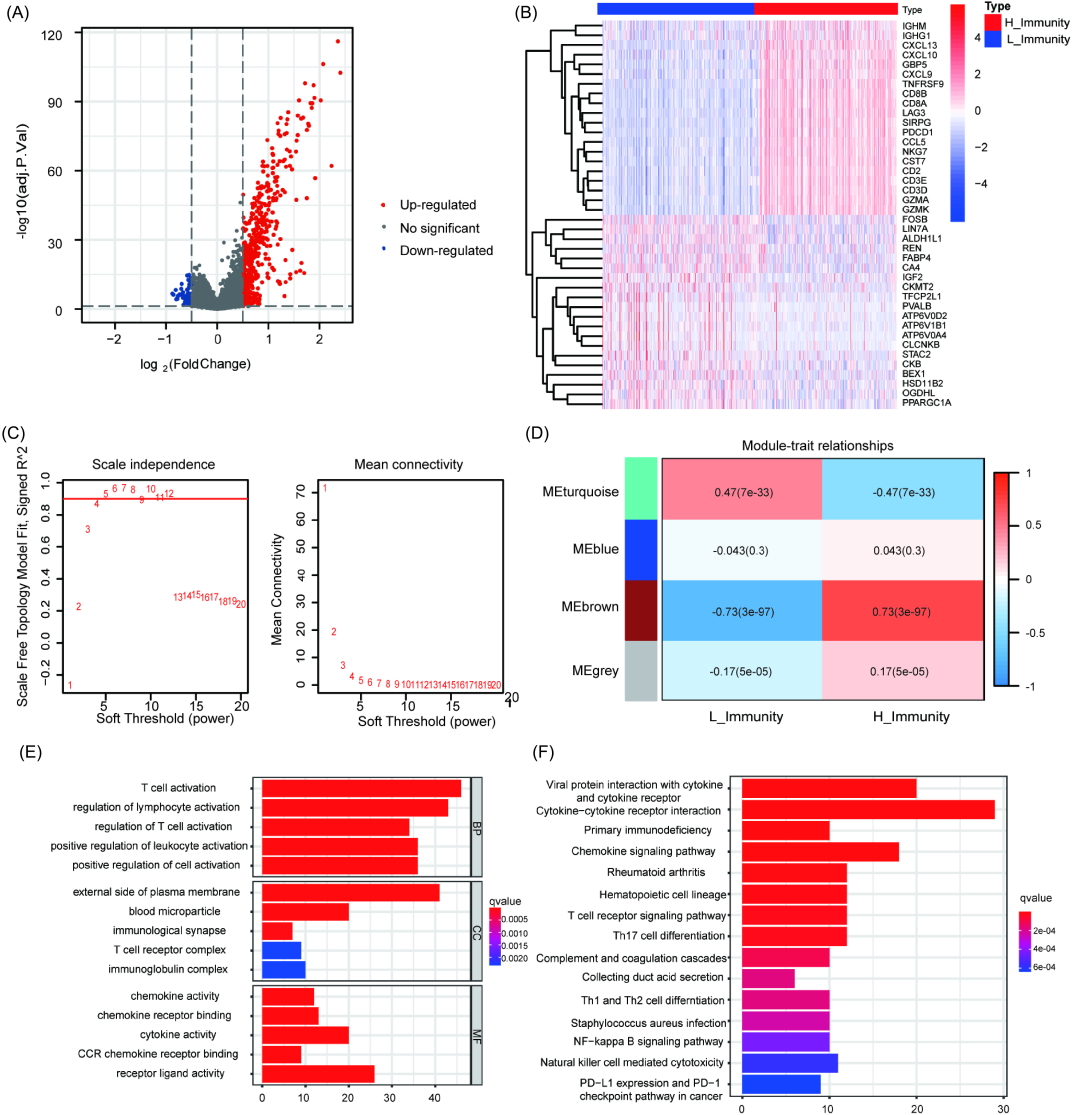
Identification of immune‐related genes. (A) Volcano plot showing that 478 upregulated genes (red) and 62 downregulated genes (blue) between L_Immunity and H_Immunity. (B) Heatmap showing the expression levels of the top 20 upregulated and downregulated genes. (C) The scale‐free fit index (left) and the mean connectivity (right). (D) Heatmap of the relationships between the state of immune and model eigengenes. (E) The results of gene ontology analysis. (F) The top 15 most significant KEGG pathways

The differentially expressed genes between H_Immunity and L_Immunity were identified according to |logFC| > 0.5 and adjusted *p* < 0.05. A total of 540 different genes were screened out, of which 478 were upregulated, and 62 were downregulated (Figure 2A,B). To identify the main module most relevant to the immune trait, WGCNA was used to further analyze these different genes. Four modules were screened according to a soft thresholding value of 4 (scale‐free *R* ^2^ = 0.87, mean connectivity = 2.26) (Figure 2C). As shown in the heatmap of the module‐trait, the brown module with 99 genes and the turquoise model with 175 genes were closely related to the immune trait (Figure 2D; *R* ^2^ = 0.73 and *p* = 3e^−97^ in the brown module, *R* ^2^ = −0.47 and *p* = 7e^−33^ in the turquoise model). Ultimately, 274 genes were regarded as IRGs. The results of GO and KEGG analyses indicated that these IRGs were closely related to T‐cell activation, regulation of lymphocyte activation, positive regulation of leukocyte activation, regulation of T‐cell activation, and other effects (Figure 2E,F).

### 3.3 Establishment and validation of a risk model

**FIGURE 3:**
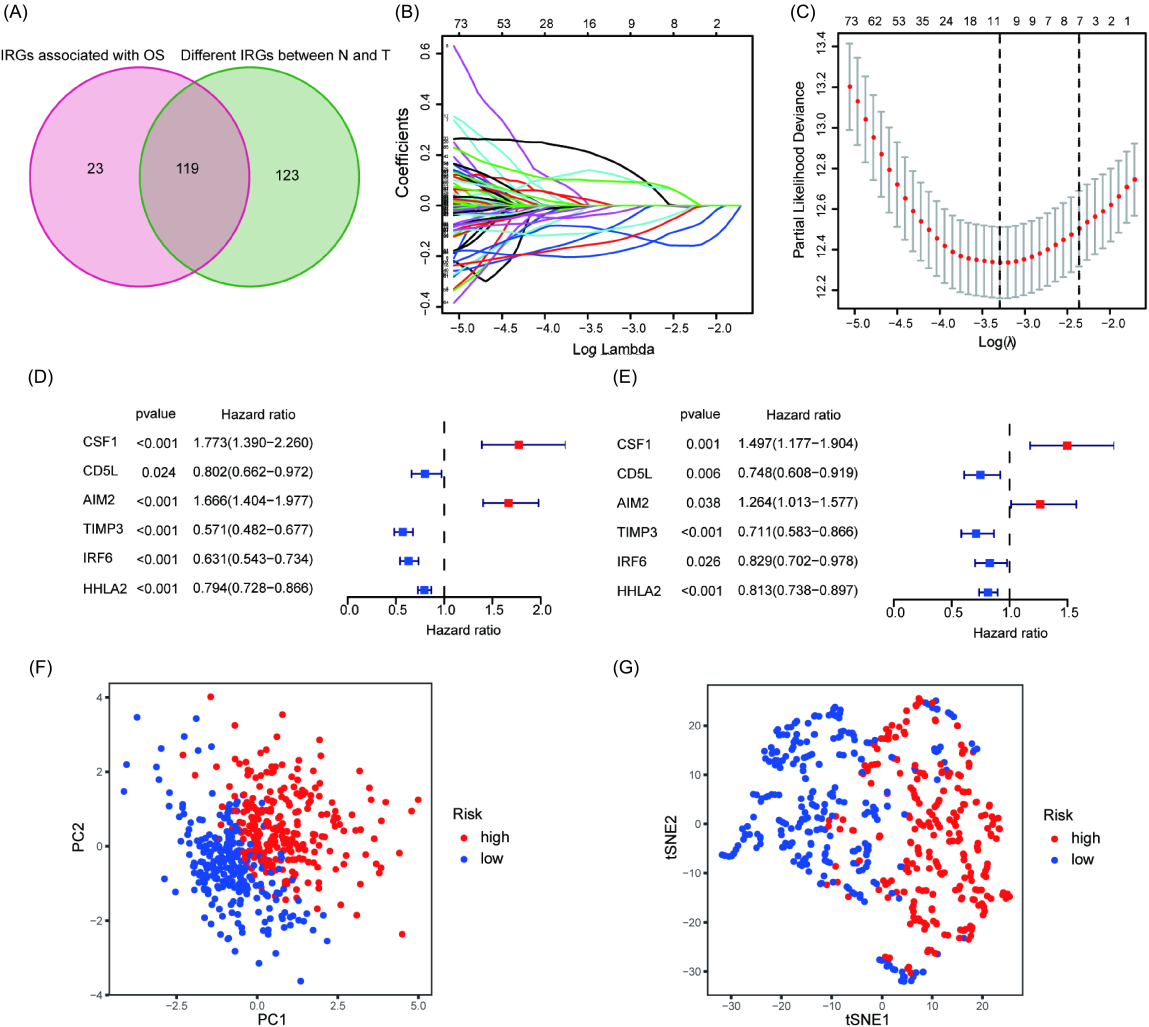
Construction of the immune‐related gene prognostic signature. (A) Venn diagram for intersect IRGs genes. (B) LASSO penalty coefficients. (C) The optimal values of the penalty parameter. Univariate (D) and multivariate (E) Cox regression analyses of the 6 genes in the risk model. (F) PCA analysis for the risk model. (G) t‐SNE analysis for the risk model

To test whether the IRGs had a potential relationship with the prognosis of ccRCC, univariate Cox regression analysis was carried out and 142 genes screened out from 274 IRGs were closely related to OS in TCGA‐KIRC. Meanwhile, 242 significantly differentially expressed IRGs between 539 ccRCC (T) and 72 normal kidney specimens (N) from TCGA‐KIRC were identified according to |logFC| > 0 and *p* < 0.05. Then, 119 intersecting genes were extracted (Figure 3A). Eleven genes screened by LASSO Cox regression analysis were further applied for multivariate Cox regression analysis (Figure 3B,C). Finally, a risk model was constructed based on six genes to assess its value in predicting the prognosis of ccRCC patients. According to the univariate and multivariate Cox regression analyses, all six genes in the risk model were found to be closely related to OS (Figure 3D,E). The risk score was estimated based on the coefficient and expression value of six genes: Risk score = (0.403 * expression of CSF1) + (−0.291 * expression of CD5L) + (0.235 * expression of AIM2) + (−0.341 * expression of TIMP3) + (−0.188 * expression of IRF6) + (−0.206 * expression of HHLA2). The samples were separated into low‐ and high‐risk groups according to the median risk score. Then, PCA and *t*‐SNE analysis were performed, and the results showed that the six‐gene signature had good performance in clustering (Figure 3F,G).

**FIGURE 4:**
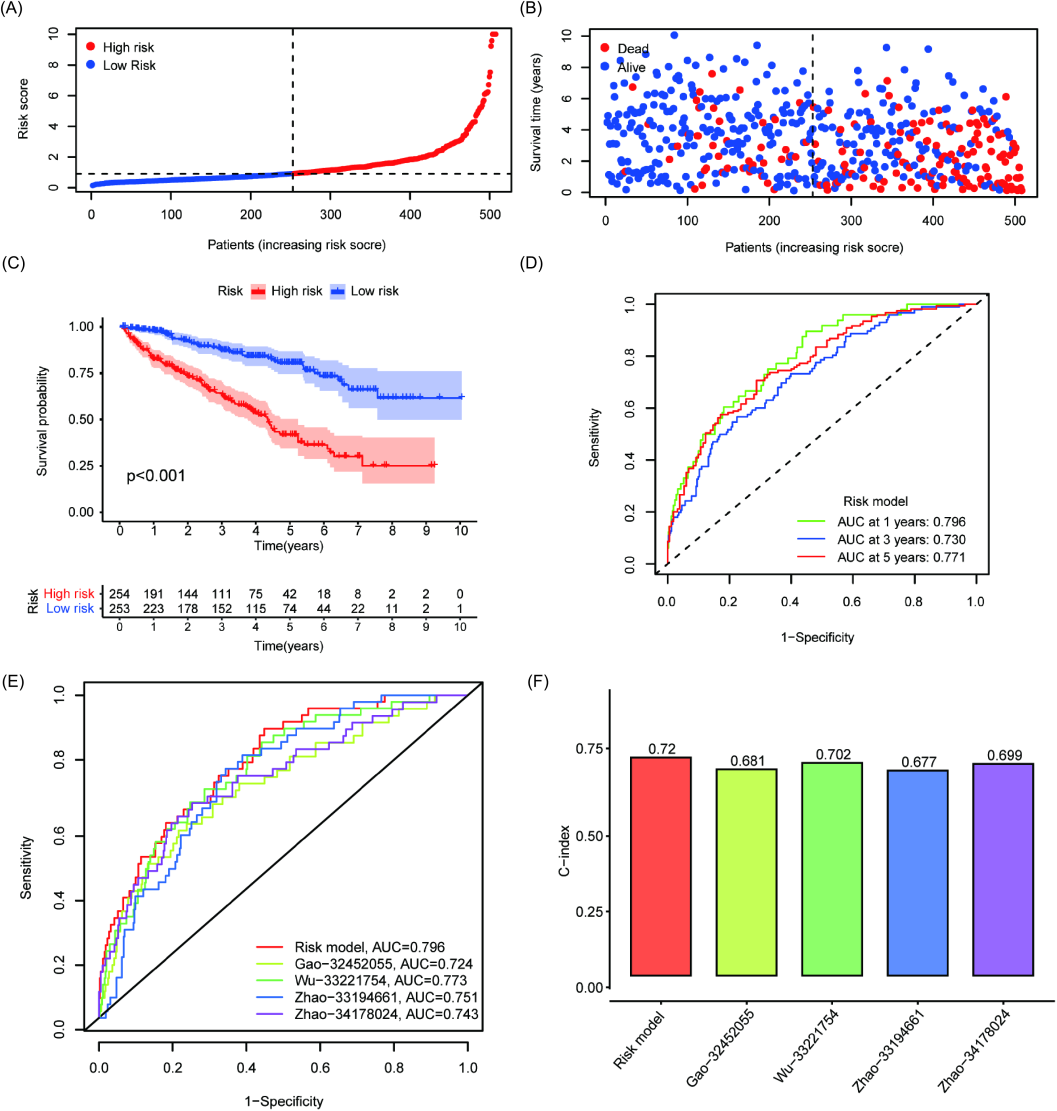
Prognostic prediction of the risk model. Distribution of risk score (A) and survival status (B). (C) Patients in the high‐risk group experienced shorter overall survival by the log‐rank test. (D) The AUC values of the 1‐, 3‐, and 5‐year ROC curves of the risk model. Comparison of the risk model with other prognostic models by ROC (E) and C‐index (F)

The distribution of the risk score and survival status of the six genes between the risk groups are shown in Figure 4A,B. Clearly, the high‐risk group contained more death samples. The OS of patients in the high‐risk group was significantly lower than that of patients in the low‐risk group (*p* < 0.001) (Figure 4C). In addition, the areas under curve (AUC) values for evaluating the predictive accuracy of the risk signature were 0.754, 0.715, and 0.739 at 1, 3, and 5 years, respectively (Figure 4D). Finally, other predictive models from different studies were used to prove whether our risk model had an advantage. The results indicated that our constructed risk model had the highest AUC and C‐index (Figure 4E,F). In conclusion, the above results suggested that the six‐gene signature had good prediction ability.

### 3.4 Relationships between the risk model and clinical characteristics

**FIGURE 5:**
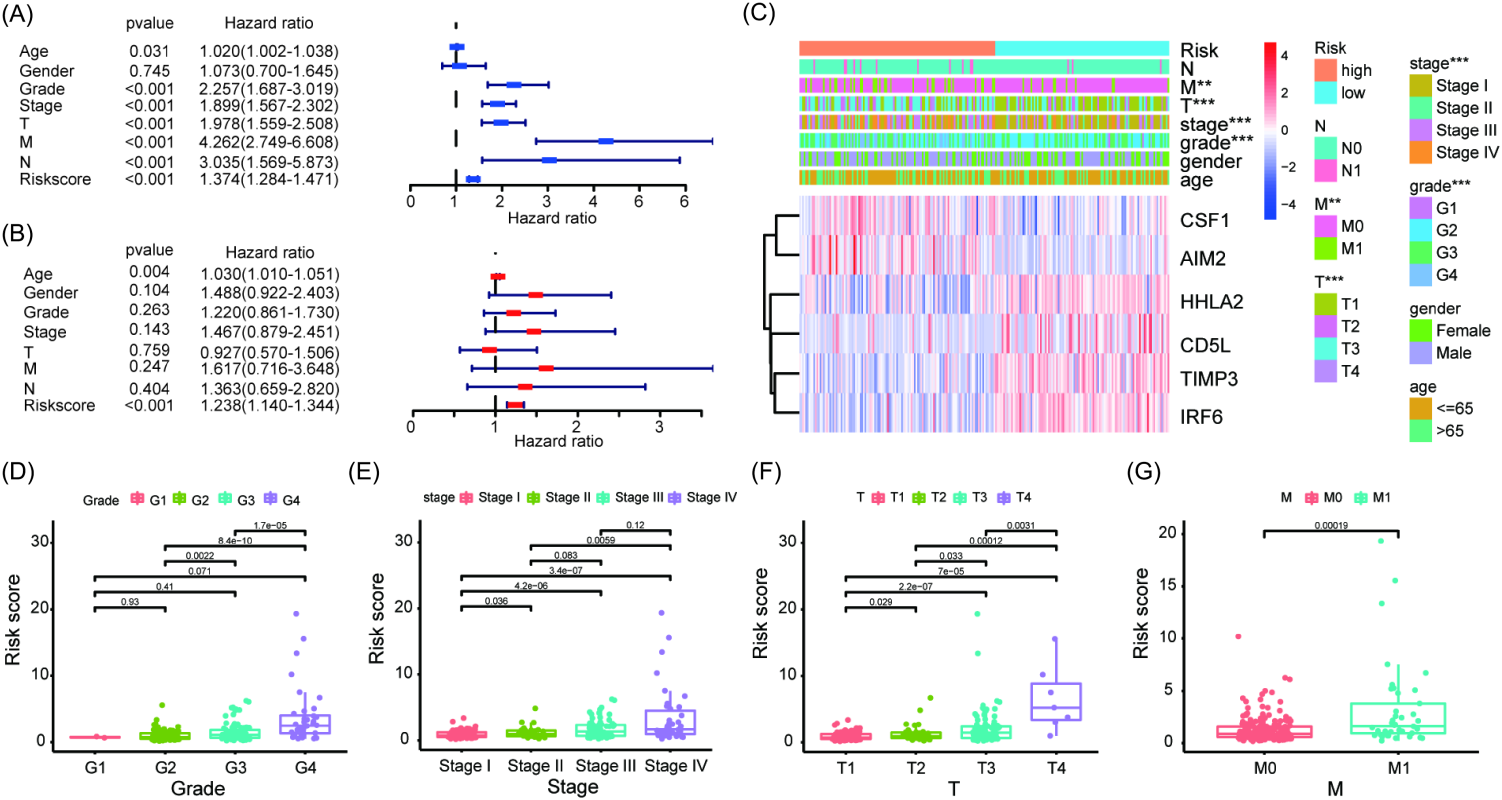
Assessment of the independent prognostic value. Univariate (A) and multivariate (B) Cox regression analyses of the risk score and clinical characteristics. (C) Distribution landscape of clinical characteristics and the expression profiles of 6 genes between the high‐ and low‐risk groups. Discrepancies in risk scores by grade (D), stage (E), T stage (F), and M stage (G). ***p < 0.001, **p < 0.01

**FIGURE 6:**
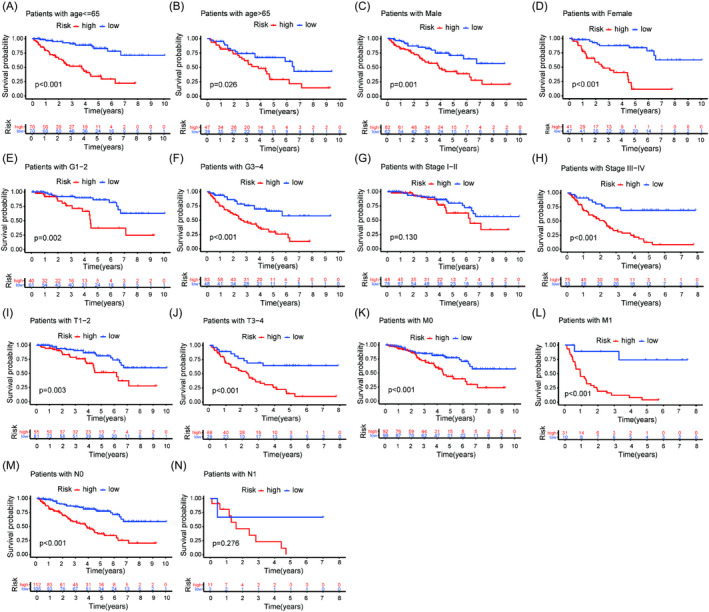
Survival analysis stratified by the clinical subgroups

To investigate whether the risk model was an independent prognostic factor, the risk score, together with other clinical characteristics, such as age, gender, grade, stage, T, N, and M were included to conduct univariate and multivariate Cox regression analyses. The results showed that the risk score was an independent factor that could be utilized to predict the prognosis of ccRCC patients (Figure 5A,B). The clinical heatmap showed that grade, stage, T, and M were closely associated with the risk by the chi‐square test (Figure 5C). The scatter diagrams also demonstrated that grade, stage, T, and M had higher risk scores by the Wilcoxon signed‐rank test (Figure 5D–G). Then, the OS stratified by the clinical subgroups between the two risk groups implied that there were significant differences among the clinical subgroups except stage I‐II and N1 (Figure 6).

### 3.5 GSEA analysis

**FIGURE 7:**
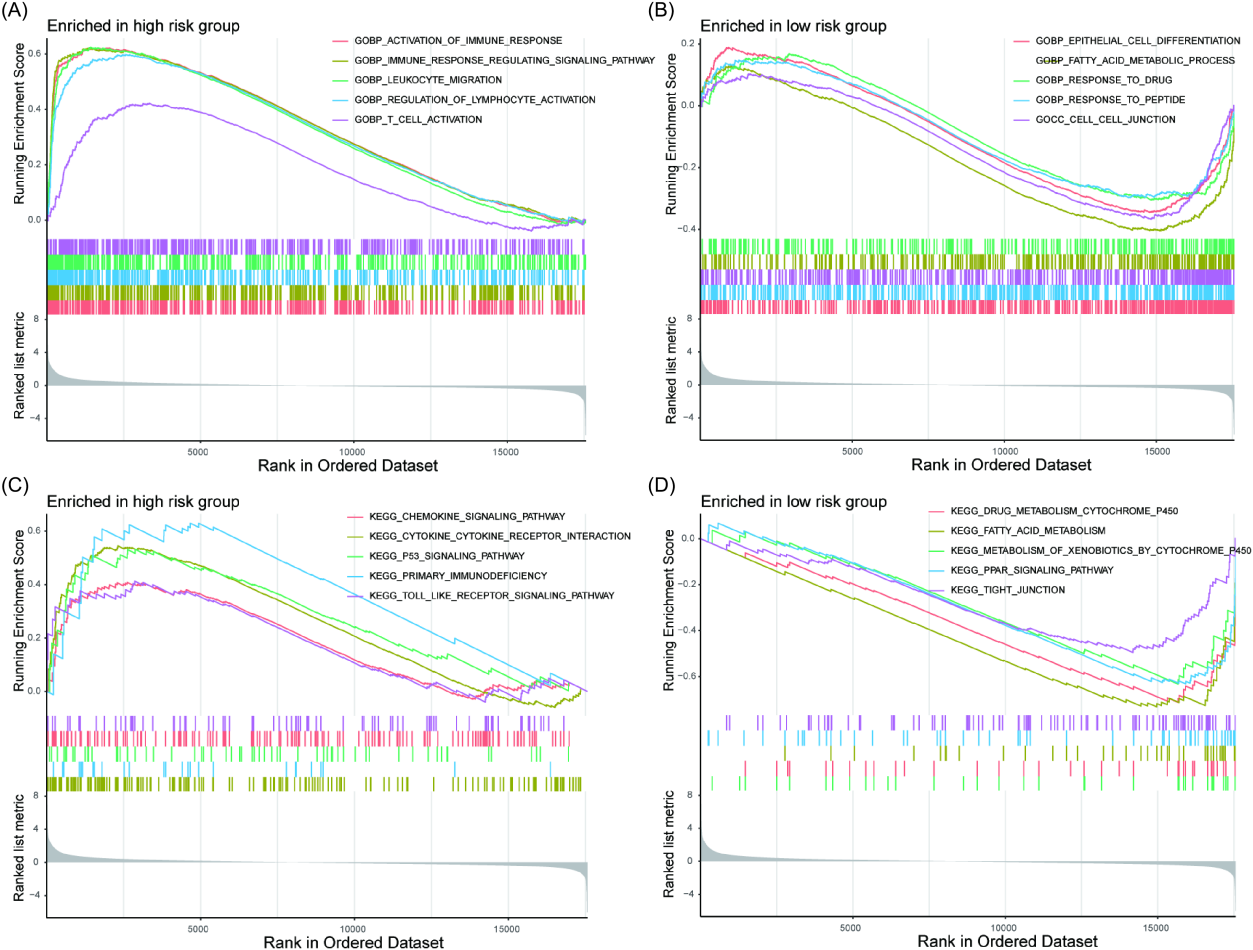
Gene set enrichment analyses between the high‐ and low‐risk groups. GO enrichment analyses in the high‐risk group (A) and the low‐risk group (B). KEGG pathway analyses in the high‐risk group (C) and the low‐risk group (D)

To illustrate the underlying mechanism of the survival difference between the two risk groups, GSEA was applied. The results of GO analysis showed that there was high enrichment of pathways in the high‐risk group, such as “activation of immune response,” “immune response regulating signaling pathway,” “leukocyte migration,” “regulation of lymphocyte activation,” and “T‐cell activation” (Figure 7A,B). Moreover, the KEGG results showed that the “chemokine signaling pathway,” “cytokine‐cytokine receptor interaction,” “p53 signaling pathway,” “primary immunodeficiency,” and “Toll‐like receptor signaling pathway” pathways were enriched in the high‐risk group (Figure 7C,D).

### 3.6 Relationship between tumor‐infiltrating immune cells and the risk model

**FIGURE 8:**
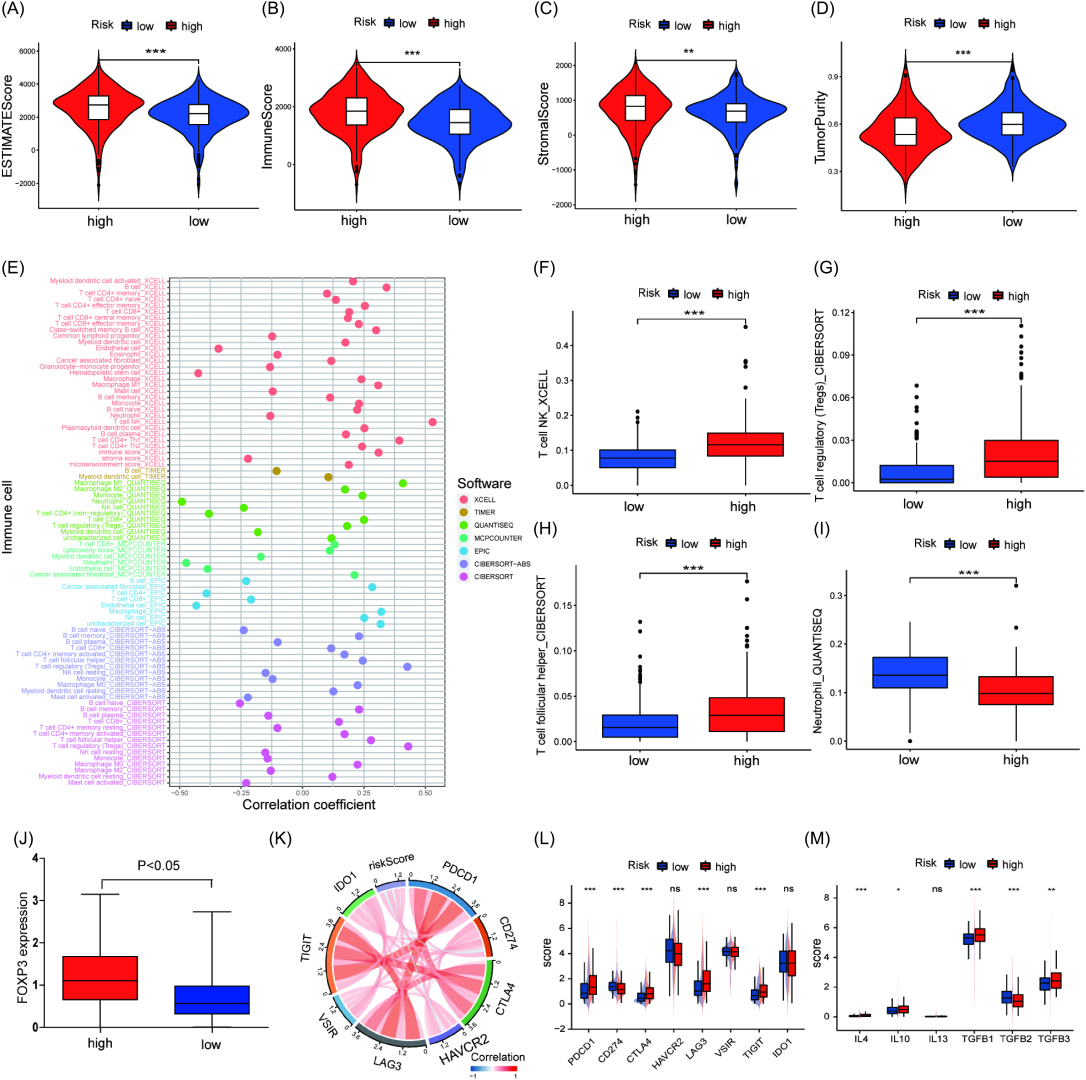
Relationship between the risk model and immunity. Differences in ESTIMATE score (A), immune score (B), stromal score (C) and tumor purity (D) between the high‐ and low‐risk groups. (E) Evaluation of the immunoinfiltrating cells by different algorithms. The number of NK T cells (F), Tregs (G) and T‐follicular helper cells (H) were higher in the high‐risk group. (I) The number of neutrophil cells were lower in the high‐risk group. (J) FOXP3 expression in the high‐ and low‐risk groups. (K) Correlation between the risk score and common immune checkpoints. (L) Expression levels of the common immune checkpoints between the high‐ and low‐risk groups. (M) Expression of the chemokines between the high‐ and low‐risk groups. ***p < 0.001, **p < 0.01

To test whether this established model could predict the tumor immune microenvironment, the results of the ESTIMATE algorithm showed that the stromal score, immune score, and ESTIMATE score in the high‐risk group were higher than those in the low‐risk group, while tumor purity had the opposite results (Figure 8A–D). Then, different methods were used to investigate the infiltration of immune cells in KIRC. The risk scores had a positive correlation with NK T cells, regulatory T cells (Tregs) and T follicular helper cells but a negative correlation with neutrophils (Figure 8E). The Wilcoxon signed‐rank test showed that the high‐risk group was characterized by the significant upregulation of NK T cells, regulatory T cells (Tregs) and T follicular helper cells, while the neutrophils had the opposite expression (Figure 8F–I). In accordance with the Treg infiltration, FOXP3 (marker for Tregs) was more highly expressed in the high‐risk group than in the low‐risk group (Figure 8G). These data indicated that the patients in the high‐risk group presented an immunosuppressive phenotype.

To confirm the immunosuppressive phenotype, common immune checkpoints and chemokines were further evaluated. The correlation analysis found that the risk scores had positive relationships with PD‐1, CTLA‐4, LAG‐3, and TIGIT, and negative relationships with PD‐L1 and HAVCR2 (Figure 8K). The histogram also indicated that the expression levels of PD‐1, CTLA‐4, LAG‐3, and TIGIT in the high‐risk group were significantly higher than those in the low‐risk group (Figure 8L). However, PD‐L1 in the high‐risk group had the opposite expression trend. Chemokines (TGF‐β, IL‐4, and IL‐10) involved in the immunosuppressive process were also significantly upregulated in the high‐risk group except TGF‐β2 (Figure 8M).^33^, ^34^, ^35^

### 3.7 The risk model was a predictive biomarker for clinical response to ICIs and targeted therapy

**FIGURE 9:**
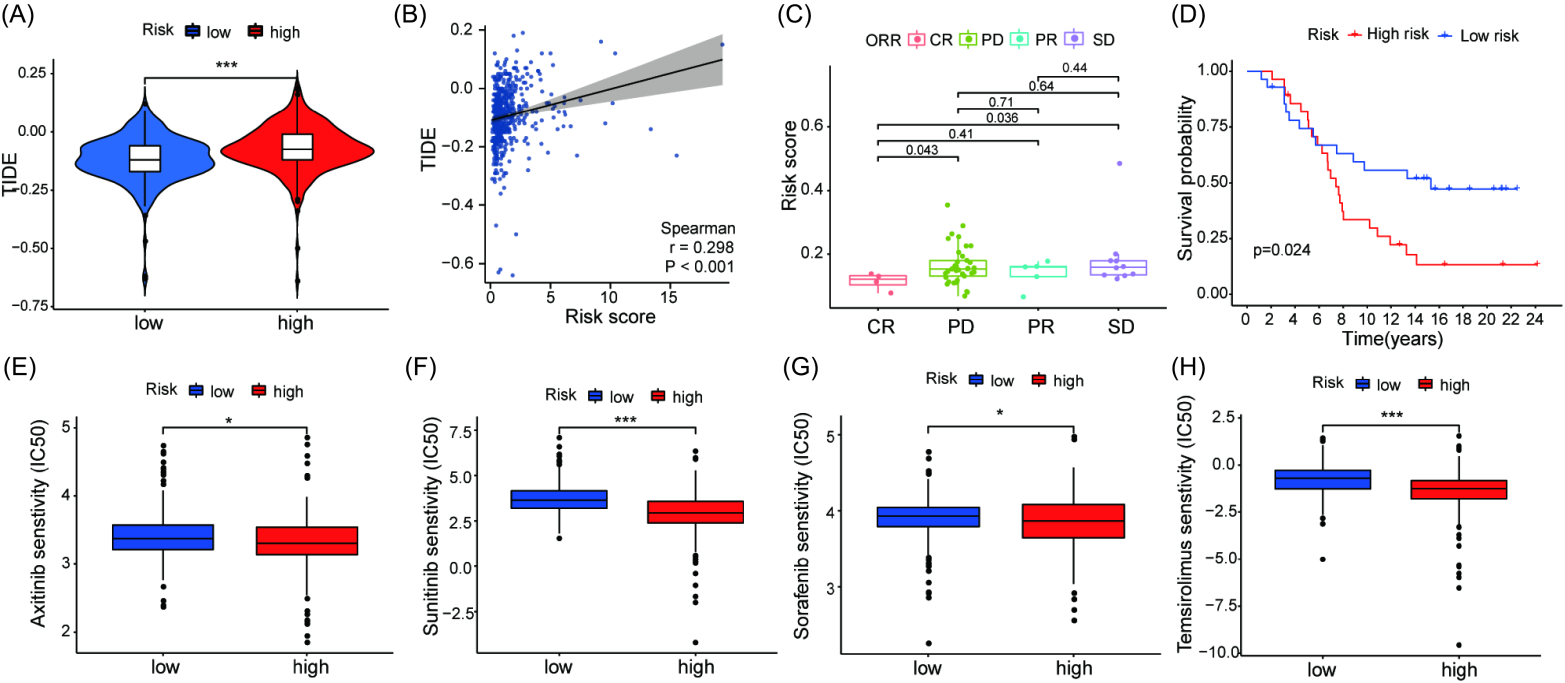
Response to immunotherapy and sensitivity to targeted therapy between the high‐ and low‐risk groups. (A) TIDE score between the high‐ and low‐risk groups. (B) Pertinence between TIDE score and risk score. (C) Relationship between risk score and response to anti‐PD‐L1 therapy in the IMvigor210 cohort. (D) OS in the high‐ and low‐risk groups after immunotherapy. IC50 values between the high‐ and low‐risk groups for axitinib (E), sunitinib (F), sorafenib (G), and temsirolimus (H). ***p < 0.001, *p < 0.05

Immune and targeted drugs have become important regimens for the treatment of advanced kidney cancer. To investigate the predictive effect of the risk model for ICIs, the TIDE method was applied to validate the response to ICIs based on the risk model. Patients in high‐risk group had higher TIDE scores than those in the low‐risk group (*p* < 0.001, Figure 9A). Moreover, there was a significantly positive correlation with the TIDE score (*r* = 0.298, *p* < 0.001, Figure 9B). Furthermore, 56 kidney cancer patients with response information in the IMvigor210 cohort were enrolled to verify the value of the risk model in predicting the response to the PD‐L1 inhibitor atezolizumab. The risk scores in patients with a complete response (CR) were significantly lower than those in patients with progressive disease (PD) and stable disease (SD) responses (*p* < 0.05, Figure 9C). Patients in the low‐risk group had significant clinical benefits and observably prolonged survival compared with those in the high‐risk group (*p* = 0.024, Figure 9D). In addition to ICIs therapy, the relationship between the risk model and the sensitivity to targeted drugs for ccRCC were further analyzed. The half inhibitory concentrations (IC50s) of antitumor drugs such as axitinib (*p* = 0.026, Figure 9E), sunitinib (*p* < 0.001, Figure 9F), sorafenib (*p* = 0.028, Figure 9G), and temsirolimus (*p* < 0.001, Figure 9H) were lower in the high‐risk group. These results demonstrated that the risk model could predict the clinical response to ICIs and targeted drug treatment in ccRCC patients.

## 4 DISCUSSION

CcRCC has been considered an immunotherapy‐responsive tumor.^36^ More recently, ICIs targeting PD‐1/PD‐L1 or CTLA‐4 have shown good clinical results among some ccRCC patients.^37^ There is no effective biomarker to predict the response to ICIs in ccRCC. Therefore, it is urgent to identify a reliable biomarker to help doctors select patients who can benefit from ICI therapy. The tumor microenvironment greatly contributes to disease biology and the response to antitumor drugs.^38^, ^39^ The immune‐inhibitor cells in the TME, such as regulatory T cells and myeloid‐derived suppressor cells, have an important role in antitumor therapy and immune escape and have become therapeutic targets for improving the efficacy of immunotherapy.^37^, ^40^ Thus, we focused on the infiltration of immune cells in the TME to construct an mRNA‐signature for predicting the prognosis and efficacy of ICIs and targeted drugs in ccRCC patients.

In this study, TCGA‐KIRC and GSE29609 mRNA expression data were merged after correcting the batch effects. Then, these samples were divided into two clusters according to 29 immune cell types and immune function by an unsupervised hierarchical clustering algorithm. The ESTIMATE algorithm and GSVA confirmed the feasibility of immune clustering. We further screened out immune‐related genes by WGCNA and built an immune‐related prognostic signature in ccRCC. ROC analysis indicated that the model was superior to common clinical characteristics and other predictive models in the prognostic prediction of ccRCC. Based on the above results, we consider that this signature has good capability for prognosis prediction in ccRCC. Subsequently, the underlying molecular mechanism of the risk model was researched. GO analysis by GSEA showed that there was high enrichment of pathways in the high‐risk group such as “activation of immune response,” “immune response regulating signaling pathway,” “leukocyte migration,” “regulation of lymphocyte activation,” and “T‐cell activation.” Moreover, KEGG results showed that “chemokine signaling pathway”, “cytokine‐cytokine receptor interaction”, “p53 signaling pathway”, “primary immunodeficiency”, and “Toll‐like receptor signaling pathway” were enriched in the high‐risk group. Therefore, immune‐related genes may participate in the progression and sensitivity to drugs of ccRCC by the above immune‐related pathways.

According to published studies, all six genes in the prognostic signature are related to immunity. CSF‐1 (colony‐stimulating factor‐1) is disproportionate in different cancers including breast, cervical, endometrial, and kidney cancers.^41^ CSF1‐secreting malignant T cells can bind to CSF1R to induce the development and survival of TAMs, promoting tumor survival and suppressing host antitumor immunity.^42^, ^43^ CD5L (CD5 molecule‐like) is a secreted glycoprotein that participates in cancer, promotes proliferation and inhibits cisplatin‐induced apoptosis in liver cancer.^44^, ^45^, ^46^ AIM2 (absent in melanoma 2) is a cytosolic innate immune receptor that has significant roles in natural immunity and inflammation by defending against exogenous and endogenous pathogens.^47^ Tissue inhibitor of metalloproteinase‐3 (TIMP3), an inhibitor of the matrix metalloproteinases (MMPs), plays a key role in regulating inflammation after injury and anticancer activity by silencing various metalloproteinases.^48^ TIMP‐3 can also induce macrophages to differentiate into proinflammatory (M1) cells.^49^ Moreover, the loss of TIMP‐3 leads to spontaneous expansion of liver CD4+ T and NKT cells.^50^ IRF6 (interferon regulatory factor 6) plays key roles in cell differentiation, regulation of immune cell development and immune responses in tumors.^51^, ^52^ HERV‐H LTR‐associating 2 (HHLA2), a member of the B7 family of immunoregulatory ligands, can mediate costimulation by interacting with transmembrane and immunoglobulin domain containing 2 (TMIGD2).^53^ The expression of HHLA2 and PD‐L1 is associated with the number of CD8(+) and CD4(+) infiltrating lymphocytes (TILs) and the poor prognosis of ccRCC patients.^54^ Blockade of both PD‐1 and HHLA2 in patients with ccRCC may be a more effective way to reverse tumor immune evasion. These results illustrate that these immune‐related genes exert their function in tumor immunology and may be new immunotherapy targets in future studies.

Furthermore, we used different algorithms to reveal the relationship between the risk model and immune cells in the TME. The patients in the high‐risk group manifested an immunosuppressive phenotype due to a higher cell abundance of infiltrating regulatory T cells (Tregs), which are immunosuppressive cells characterized by the expression of FOXP3.^55^ Tregs can suppress immune activation by secreting immune‐suppressive cytokines (IL‐10, IL‐35, and TGF‐β) or expressing coinhibitory molecules such as CTLA‐4, PD‐1, LAG‐3, and TIGIT.^56^, ^57^, ^58^ In our study, Tregs had high expression in the high‐risk group. Meanwhile, cytokines (IL‐4, IL‐10, IL‐13, TGF‐β1, and TGF‐β3) and checkpoints (CTLA‐4, PD‐1, LAG‐3, and TIGIT) involved in immune suppression were highly expressed in the high‐risk group, which was attributed to the infiltration of Tregs. These results imply that our risk signature has the potential to predict infiltrating immune cells in ccRCC, which might be beneficial for the immunotherapy. We further studied the relationship between the risk model and the response to immunotherapy by the TIDE algorithm, which has been used to predict the therapeutic response to ICIs.^30^ Notably, the TIDE score in the high‐risk group was higher than that in the low‐risk group, which indicated an undesirable response to immunotherapy due to more T‐cell dysfunction or more exclusion of T‐cell infiltration. Furthermore, the analysis based on the IMvigor210 cohort also demonstrates that patients with low‐risk scores have a better response to ICIs. Additionally, the inhibitory concentration (IC50) values of axitinib, sunitinib, sorafenib, and temsirolimus were lower in the high‐risk group than in the low‐risk group, which signified that the patients in the high‐risk group were more sensitive to these drugs. Collectively, the risk model could contribute to the prediction of ccRCC patients’ response to immunotherapy and targeted drugs.

Some limitations are presented in the study. First, there are no external data to validate the predictive ability of the risk model. Second, clinical and laboratory studies are needed to confirm the value of the risk model in clinical applications.

In summary, we constructed a risk signature based on the six immune‐related genes that could act as an independent prognostic factor and had a reliable predictive value in the immunotherapy response and targeted drug sensitivity of ccRCC patients.

## CONFLICT OF INTEREST

The authors declared no conflicts of interest.

## AUTHOR CONTRIBUTIONS

Libin Zhou was responsible for study conception and design, data acquisition, data analysis, and drafting and revision of the study. Hualong Fang was responsible for data analysis and drafting. Min Yin and Huimin Long were responsible for data acquisition and revision of the study. Guobin Weng was responsible for revision of the study.

## Data Availability

The data that support the findings of this study are available from the corresponding author upon reasonable request.
